# Lead Exposure Causes Spinal Curvature during Embryonic Development in Zebrafish

**DOI:** 10.3390/ijms23179571

**Published:** 2022-08-24

**Authors:** Xueting Li, Ce Chen, Mingyue He, Lidong Yu, Renhao Liu, Chunmeng Ma, Yu Zhang, Jianbo Jia, Bingsheng Li, Li Li

**Affiliations:** 1 School of Life Science and Technology, Harbin Institute of Technology, Harbin 150080, China; 2 School of Physics, Harbin Institute of Technology, Harbin 150080, China; 3 State Key Laboratory of Environmental Aquatic Chemistry, Research Center for Eco-Environmental Sciences, Chinese Academy of Sciences, Beijing 100085, China; 4 University of Chinese Academy of Sciences, Beijing 100049, China; 5 School of Biotechnology and Health Sciences, Wuyi University, Jiangmen 529020, China; 6 Key Laboratory of UV Light Emitting Materials and Technology of Ministry of Education, Northeast Normal University, Changchun 130024, China

**Keywords:** Pb^2+^, toxicology, endoplasmic reticulum stress, apoptosis, somatic segment development

## Abstract

Lead (Pb) is an important raw material for modern industrial production, they enter the aquatic environment in several ways and cause serious harm to aquatic ecosystems. Lead ions (Pb^2+^) are highly toxic and can accumulate continuously in organisms. In addition to causing biological deaths, it can also cause neurological damage in vertebrates. Our experiment found that Pb^2+^ caused decreased survival, delayed hatching, decreased frequency of voluntary movements at 24 hpf, increased heart rate at 48 hpf and increased malformation rate in zebrafish embryos. Among them, the morphology of spinal malformations varied, with 0.4 mg/L Pb^2+^ causing a dorsal bending of the spine of 72 hpf zebrafish and a ventral bending in 120 hpf zebrafish. It was detected that spinal malformations were mainly caused by Pb^2+^-induced endoplasmic reticulum stress and apoptosis. The genetic changes in somatic segment development which disrupted developmental polarity as well as osteogenesis, resulting in uneven myotomal development. In contrast, calcium ions can rescue the series of responses induced by lead exposure and reduce the occurrence of spinal curvature. This article proposes new findings of lead pollution toxicity in zebrafish.

## 1. Introduction

The heavy metal Pb is widely used in manufacturing as well as metallurgy, and with the variety of lead-containing wastes, it has become one of the most prevalent and toxic environmental pollutants. Although lead emissions have been reduced to a great extent globally, the impact of trace amounts of lead cannot be underestimated [1]. The hazard of trace amounts of lead to living organisms has received continuous attention. Studies have shown that Pb can cause damage to the human nervous system, hematopoietic system, digestive system, cardiovascular system, urinary system, reproductive system and immune system [2,3]. Heavy metals have been shown to compromise antioxidant activity. Lead forms a complex with glutathione, inhibits glutathione peroxidase, and interferes with oxidative phosphorylation. Saturation or compromise of these antioxidant processes results in accumulation of ROS and damage to macromolecules (proteins, lipids, and nucleotides), failure in cell function and death [4]. In some cases, very low doses of Pb can cause irreversible damage to the human body. Spinal malformations have a high environmental sensitivity [5] and are one of the common diseases caused by the long-term accumulation of heavy metals in living organisms. Witeska [6] concluded that heavy metals in living organisms first accumulate in bone tissue, leading to a decrease in Ca and P content causing changes in bone mechanical properties, including body and skeletal deformation.

Zebrafish have high reproductive rates, distinct developmental features, transparent embryos for easy observation, and a high degree of homology with advanced vertebrates in genetics and embryonic development [7]. Therefore, zebrafish has been widely used for toxicological assessment of heavy metals, drugs and organic pollutants [8]. Through a previous study, we found that lead-treated zebrafish produced severe spinal curvature, which is consistent with previous findings [9]. However, there is no convincing explanation for the mechanism by which Pb^2+^ cause spinal curvature. Here, we further explain the cause of spinal curvature in developing zebrafish due to Pb^2+^ by studying different time points, different lead ion concentrations, and the addition of rescue drugs. This study provides new ideas on the causes of spinal curvature in developing zebrafish due to lead exposure, and provides a scientific basis for evaluating the hazard of Pb^2+^ to aquatic organisms and guiding the monitoring of lead pollution in water bodies.

## 2. Results

### 2.1. Toxicity of Pb^2+^ to Zebrafish Embryos

**Figure 1:**
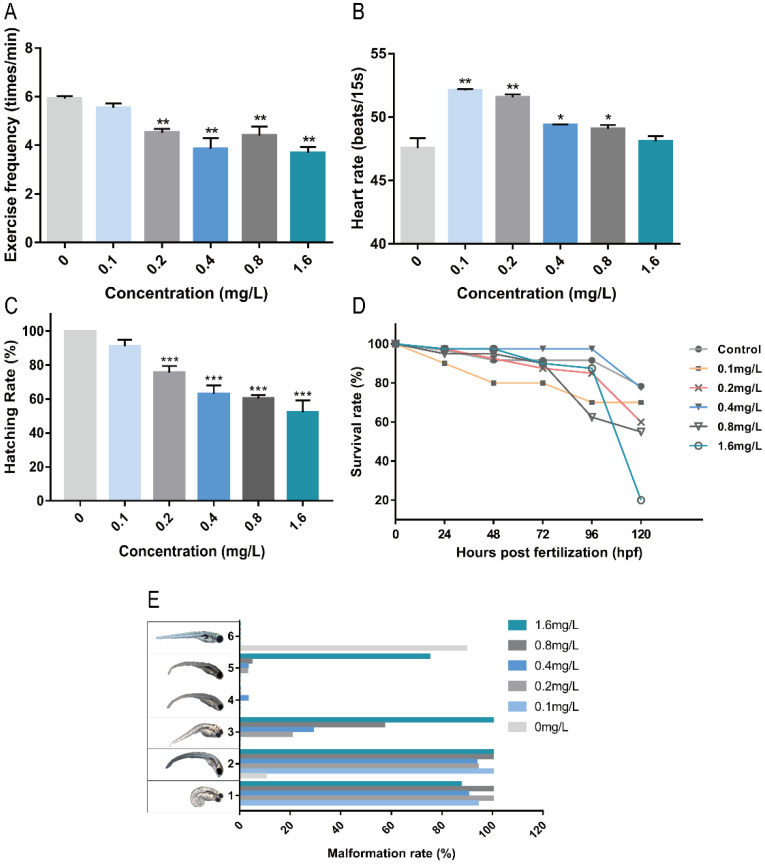
The effects of different concentrations of Pb^2+^ on zebrafish embryos. (**A**) 24 hpf exercise frequency; (**B**) 48 hpf heart rate; (**C**) 57 hpf hatching rate; (**D**) 120 hpf survival rate; (**E**) 120 hpf Malformation rate (1, spinal curvature; 2, absence of swim bladder; 3, pericardial cavity edema; 4, pigment deficiency; 5, cerebral hemorrhage; 6, normal juvenile zebrafish). (* *p* < 0.05, ** *p* < 0.01, *** *p* < 0.001, *n* = 15, repeat three times).

Exercise frequency is an important index of zebrafish nervous system development. The frequency of exercise can reflect the stimulation or damage to the nervous system of zebrafish. As shown in Figure 1A, Pb^2+^ at concentrations higher than 0.2 mg/L significantly reduced the level of exercise frequency in zebrafish embryos, indicating some effects on the nervous system of zebrafish. The heart of zebrafish was basically developed by 48 hpf, and the blood circulation system was gradually established [10]. As shown in Figure 1B, Pb^2+^ (0.1–0.8 mg/L) caused heart rate acceleration. Low concentrations of Pb^2+^ (0.1 mg/L and 0.2 mg/L) significantly increased the heart rate and the stimulation to the heart was the most obvious, indicating some effect on the early development of the heart and the circulatory system in zebrafish. As shown in Figure 1C, the hatching rate decreased significantly with increasing Pb^2+^ concentration in a concentration-dependent manner. Delayed hatching occurred in the high concentration group. It indicates that Pb^2+^ delays the developmental period of zebrafish embryos.

The mortality rate reflects the acute toxicity of lead ions to zebrafish embryos. The cumulative survival rate of each group within 120 hpf was recorded. As shown in Figure 1D, the cumulative survival rates of zebrafish embryos were reduced under the exposure of Pb^2+^. The survival rate of low concentration of Pb^2+^ was higher. The survival rate of low concentration of Pb^2+^ was higher. The highest survival rate was 80% in the 0.4 mg/L group, while the highest concentration of 1.6 mg/L group survived only 20%, indicating that high concentrations of lead ions directly affected the survival of zebrafish. Subsequently, we observed the surviving zebrafish malformations and recorded five common malformations at 120 hpf, as shown in Figure 1E. The overall malformation rate was found to be 100% in each Pb^2+^ experimental group. The main malformations were curvature of the spine and absence of the swim bladder, and there was a co-occurrence of both. Some other malformations were observed in the Pb^2+^ group except for the 0.1 mg/L Pb^2+^ group. The incidence of pericardial cavity edema increased with increasing concentration in a concentration-dependent trend. In addition, a large number of brain hemorrhages were observed in the 1.6 mg/L group, and a unique pigment deficiency was observed in the 0.4 mg/L group. The above results indicate that both low and high concentrations of lead ions are toxic to zebrafish embryos, affecting cardiovascular and neurological development, leading to malformations and even death.

**Figure 2:**
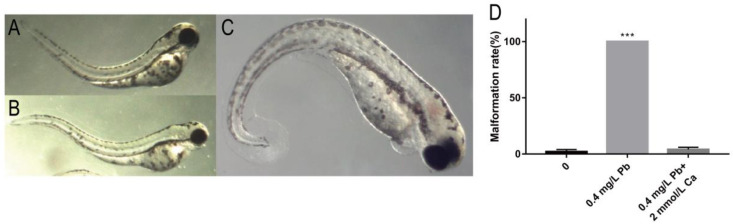
Spinal malformation in zebrafish embryos at different periods of 0.4 mg/L Pb^2+^ exposure. (**A**) Bend upwards at 72 hpf; (**B**) S-shaped curved at 96 hpf; (**C**) Bend downward at 120 hpf; (**D**) Malformation rate at 120 hpf. (*** *p* < 0.001, *n* = 50, Repeat three times).

Based on the results of mortality and malformation rate, we found that the survival rate of 0.4 mg/L Pb^2+^ solution was high, and the malformation rate of spinal curvature was high at 120 hpf. Therefore, we chose 0.4 mg/L Pb^2+^ to study the reason of causing spinal curvature. The results showed that the spinal curvature caused by Pb^2+^ started at 72 hpf, and the type of malformation was upward curvature, with 52% of juvenile zebrafish showing upward curvature (Figure 2A). At 96 hpf, the main malformation changed to S-shaped curvature, with 85% of juvenile zebrafish showing S-shaped curvature (Figure 2B). At 120 hpf, the malformation changed to mainly downward curvature, with 100% of zebrafish showing downward curvature (Figure 2C). The specific statistical results are shown in Table 1.

It has been reported that calcium phosphate salts can be used as rescue drugs for Pb exposure [11]. Zebrafish embryos co-treated with 2.0 mmol/L calcium ions (Ca^2+^) and 0.4 mg/L Pb^2+^ showed almost the same malformation rate as the control group, indicating the elimination of the effect of Pb^2+^ at 120 hpf (Figure 2D).

In this study, the zebrafish spinal curvature model is established using Pb^2+^. Based on the statistics of malformation and lethality, it is clear that Pb^2+^ caused deformities such as curvature of the spine, and high concentrations have lethal effects, which is consistent with previous studies. From the statistics of malformation rate, it is found that the deformation of swim bladder and spinal curvature are more likely and concurrent. It is found that Pb^2+^ slows down the bone development of zebrafish embryos and causes irregularities in the musculature. Pb^2+^ can cause the loss of calcium ions in the body. The toxicity caused by lead ions can be mitigated by calcium ion rescue experiments.

### 2.2. The Mechanism of Spinal Curvature

Calcein binds Ca^2+^ in tissues and will appear yellow-green under fluorescence. It does not have a large effect on zebrafish and can be used as a good dye for in vivo staining of skeletal development to detect the degree of skeletal calcification in juvenile zebrafish. After staining, hard bones will appear yellow-green, and tissues containing calcium such as muscles can be seen clearly under the fluorescence microscope. This is a very convenient use for observing bones as well as muscle morphology.

**Figure 3:**
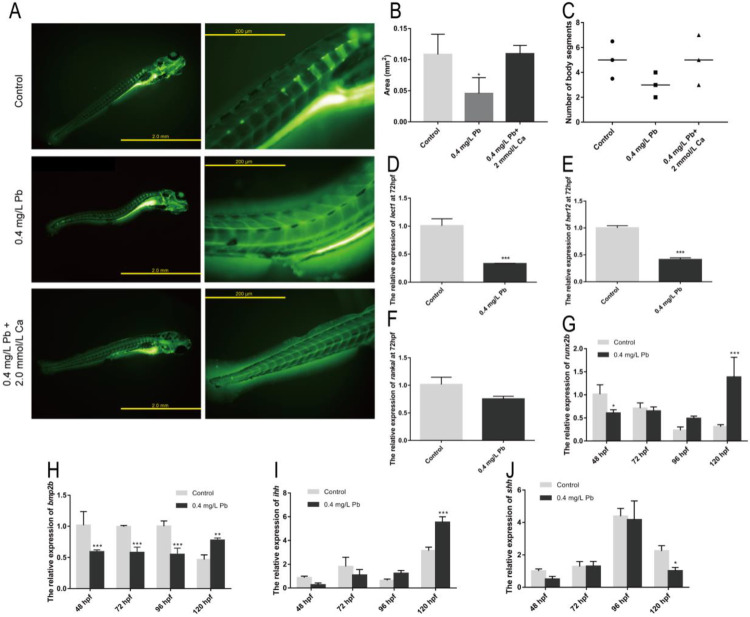
Skeletal and somites development. (**A**) Calcein staining; (**B**) Area of ossification in juvenile zebrafish; (**C**) Body segment statistics. The expression of the somites development-related genes is shown for (**D**) *lect1,* (**E**) *her12*, (**F**) *rankal* (**G**) *runx2b*, (**H**) *bmp2b*. The expression of Hedgehog signaling pathway gene of zebrafish embryos is shown for (**I**) *ihh* and (**J**) *shh*, with *ef1α* as reference. (* *p* < 0.05, ** *p* < 0.01, *** *p* < 0.001, *n* = 50).

Calcein staining was performed on zebrafish at 120 hpf to observe the skeletal ossification and somites development (Figure 3A). It was found that the ossification area of the Pb^2+^ group (0.4 mg/L) was smaller than that of the control group and the calcium rescue group (Figure 3B), and the number of somites was also smaller than that of the other two groups (Figure 3C). In addition, the Pb^2+^ group (0.4 mg/L) showed an uneven distribution of sarcomeres at the site of spinal curvature, showing shortened sarcomeres at one end and longer sarcomeres at the other, which differed significantly from the regular herringbone sarcomeres of the control group (Figure 3A). This suggests that Pb^2+^ contributes to spinal curvature in zebrafish by affecting sarcomeres development and bone formation. Subsequently, we look for the reason at the gene level.

*lect1* (chondromodulin) is expressed in cartilage and skull structures [12]. *her12* (hairy-related 12) is involved in the Notch signaling pathway and regulates nervous system development [12]. *rankal* is expressed in head and scale, regulating osteoblast differentiation [12]. Bone morphogenetic protein 2b (*bmp2b)* [13] and RUNX family transcription factor 2b (*runx2b)* [14] together promote osteoblast maturation and differentiation. These genes play an important role in the development of the zebrafish skeletal system and represent the degree of development of the skeletal system. The expression of *lect1* and *her12* was significantly down-regulated after Pb^2+^ treatment, and rankal also showed a down-regulation trend (Figure 3D–F). This demonstrates that Pb^2+^ has serious effects on zebrafish somites and skeletal development. During osteoblast formation, *bmp2b* and *runx2b* were also significantly down-regulated (Figure 3G,H). The decreased expression levels of *lect1*, *her12*, *bmp2b* and *runx2b* together affect the skeletal and somatic segment development in zebrafish. This may be one of the important reasons for the spinal curvature.

In addition, we found that zebrafish with spinal curvature caused by Pb^2+^ were often accompanied by swim bladder deficiency. There is a linkage relationship between these two deformities. The Hedgehog signaling pathway is an important pathway that controls swim bladder development [15]. As shown in Figure 3I, the expression of Indian hedgehog (*ihh*) gene was not significantly different from the control until 96 hpf. However, this gene was significantly upregulated at 120 hpf. Meanwhile, the Sonic hedgehog (*shh*) gene was significantly decreased at 120 hpf (Figure 3J), indicating that Pb^2+^ affected swim bladder development through the Hedgehog pathway. Changes in the expression levels of *ihh* and *shh* may affect developmental polarity leading to the appearance of spinal curvature [16]. The above results suggest that Pb^2+^ regulates the development of zebrafish somites mainly through *lept1* and *her12*, inhibits skeletal development in zebrafish through *bmp2b* and *runx2b*, and affects the process of skeletal and swim bladder development through *ihh* and *shh*.

### 2.3. Pb^2+^ Induce Apoptosis by Activating Endoplasmic Reticulum (ER) Stress

**Figure 4:**
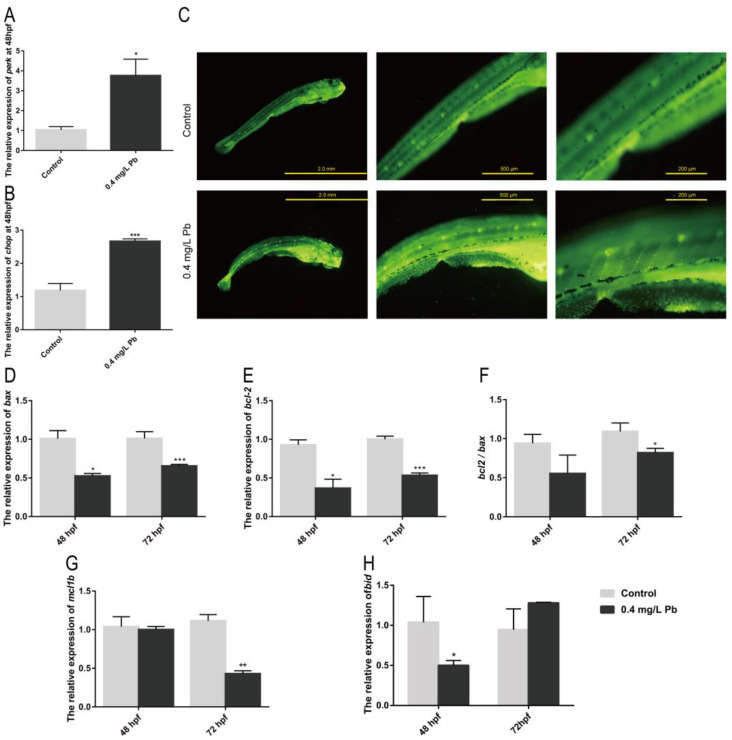
mRNA expression of ER stress and apoptosis-related genes in zebrafish. mRNA expression of ER stress-related genes in zebrafish embryos at 48 hpf using ef1α as reference: (**A**) *perk*, (**B**) *chop*. (**C**) AO staining; mRNA expression of apoptosis-related genes in zebrafish embryos at 48 and 72 hpf; (**D**) *bax*; (**E**) *bcl-2*; (**F**) *bcl-2/bax* ratio; (**G**) *mcl1b*; (**H**) *bid*. (* *p* < 0.05, ** *p* < 0.01, *** *p* < 0.001, *n* = 50.)

Current studies have shown that oxidative damage is one of the important causes of organismal damage caused by Pb^2+^. It is also reported that the ER stress pathway is also involved in the regulation of *runx2b*, a gene related to bone development. Among them, *perk/chop* are important genes involved in ER stress, and they can regulate *runx2b* with osteoblast differentiation and maturation. It was found that both *perk* and *chop* were significantly upregulated in the Pb^2+^-treated group compared with the control group at 48 hpf, as shown in Figure 4A,B. Therefore, it is demonstrated that Pb^2+^ induce ER stress in zebrafish embryos. Activation of ER stress chop signaling pathway triggers apoptosis.

In apoptotic cells, apoptotic bodies are formed due to chromatin consolidation or breakage into pieces of varying sizes. Acridine orange stained them with yellow-green fluorescence to detect apoptotic cells, as shown in Figure 4C. It was found that a large amount of green fluorescence appeared in the zebrafish muscular segment part in the Pb^2+^ experimental group. This indicates that Pb^2+^ promotes the occurrence of apoptosis in zebrafish muscle cells. Combined with the previous deformation and uneven distribution of muscle nodes, it suggests that abnormal muscle cell development and apoptosis may be closely associated with their spinal curvature.

One of the key indicators of apoptosis is whether the ratio of anti-apoptotic gene *bcl-2* to the pro-apoptotic gene *bax* is down-regulated. The expression of *bcl-2* and *bax* genes was detected by RT-PCR (Figure 4D,E). Both genes showed significant down-regulation at 48 hpf as well as 72 hpf relative to the control group. Additionally, at 72 hpf, the *bcl-2* and *bax* ratios appeared significantly down-regulated (Figure 4F). This indicates that apoptosis occurred at 72 hpf. Meanwhile the anti-apoptotic gene *mcl1b* was significantly decreased at 72 hpf, and the pro-apoptotic gene *bid* was increased (Figure 4G,H). It further demonstrated that Pb^2+^ promoted apoptosis in myocytes.

## 3. Discussion

Studies have shown that Pb^2+^ increases oxidative stress levels, causing endoplasmic reticulum stress early in development [3]. Pb^2+^ trigger ER stress, which is involved in protein folding and calcium homeostasis. ER stress leads to abnormal endoplasmic reticulum function, triggering apoptosis and inflammation [17]. The molecular chaperone of PERK, BIP, is one of the most abundant proteins in the ER. PERK is considered a major sensor of the unfolded protein response, and BIP separates from PERK when the ER stress response is received, activating PERK to cause apoptosis [18]. CHOP is considered a marker protein to promote apoptosis [19]. The increase in PERK and CHOP in this study indicates that the ER stress response activates the onset of apoptosis.

On the one hand, the accumulation of Pb^2+^ in vivo triggers apoptosis, which deforms the musculature and leads to spinal curvature. On the other hand, Pb^2+^ stimulates the Hedgehog signaling pathway, causing the downward curvature of the spine. During endochondral ossification, secreted signal, Ihh has been shown to regulate the onset of hypertrophic differentiation of chondrocytes. BMPs, family of secreted factors regulating bone formation, have been implicated as potential interactors of the IHH [20]. Functional integration or Synergistic regulation between Hedgehog signaling pathway and BMPs signaling pathway promotes ALP expression and osteogenic differentiation [21]. In addition, Pb^2+^ affects the formation of bones and muscle nodes by suppressing the notochord-associated *shh.* It is because lead ions can lead to the loss of bone calcium [22]. We suppose that the cause of Pb^2+^-induced spinal curvature is the loss of calcium in vivo, which is caused by the competition between Pb^2+^ and Ca^2+^. The reduced activity of calcium-binding calmodulin protein causes downregulation of the *shh*, which affects *bmp*, causing changes in *ihh*, *runx2b*, and leading to spinal curvature.

This study provides two possible mechanisms of Pb^2+^-induced spinal curvature, namely, endoplasmic reticulum stress–apoptosis pathway and bone development-related gene regulation pathway. Both exhibit obvious changes, but which one is the former needs further research. It provides a scientific basis for evaluating the hazard of Pb^2+^ to aquatic organisms and guiding the monitoring of lead pollution in water bodies.

## 4. Materials and Methods

### 4.1. Materials and Reagents

Lead nitrate, calcium phosphate, Tricaine and paraformaldehyde used in the experiments were purchased from Biopped, Paterson, NJ, USA. Alizarin red, calcein and acridine orange were purchased from Solarbio Co., Ltd. (Beijing, China). Trizol was purchased from TaKaRa, Kyoto, Japan.

### 4.2. Breeding and Spawning of Zebrafish

The AB strain zebrafish used in this experiment was obtained from the Heilongjiang Fisheries Research Institute under the Chinese Academy of Fisheries Science, and was raised in our laboratory for more than two months. The water temperature was 28 °C, and the light/dark cycle was 14 h/10 h. The food was fed twice a day, at 9:00 and 15:00, with live *Artemia* cysts (Beijing, China).

### 4.3. Zebrafish Pb^2+^ Exposure Experiment

Deionized water (DW) was used to wash fertilized eggs and then transferred to 24-well cell culture plates for Pb^2+^ exposure experiments. The maximum allowed discharge concentration of Pb^2+^ is 1.0 mg/L according to the National Comprehensive Discharge Standard GB 8978-1996 of the People’s Republic of China. Therefore, this experiment used lead nitrate at concentrations of 0.1 mg/L, 0.2 mg/L, 0.4 mg/L, 0.8 mg/L and 1.6 mg/L according to the national standard concentration (1.0 mg/L), with deionized water as the control. Three parallel replicates were set up with 45 fertilized eggs per group using DW as control. The culture plates were placed in a biochemical constant-temperature incubator, at a constant 28 °C. The culture medium was changed every 24 h during the experiment, and dead individuals were removed.

The frequency of exercise was observed at 24 hpf, the heart rate was measured for 15 s at 48 hpf, and the hatching rate was counted at 54 hpf. The number of surviving individuals was recorded every 24 h during the entire cycle of the experiment. Hatched larvae were anesthetized with Tricaine and fixed in 4% paraformaldehyde. Photographs and statistics of deformities were taken with stereomicroscope.

### 4.4. Alizarin Red Staining

Zebrafish embryos were fixed with 4% PFA for 2 h, rinsed twice with PBS, bleached with 1.5% H_2_O_2_/1% KOH for 5 min, rinsed twice with 25% glycerol/0.1% KOH for 20 min, stained with 0.05% alizarin red for 30 min avoiding light, rinsed twice with 50% glycerol/0.1% KOH, and stored at 4 °C with 50% glycerol/0.1% KOH.

### 4.5. Calcein Staining

Zebrafish embryos were added with 0.2% calcein solution, kept in the dark for 1 h, washed with E3 solution and left for 2 h to release the excess dye in the embryos, and washed twice with PBS. After zebrafish embryos were anesthetized with 0.05% Tricaine for 5 min, the development of fish skeleton was observed and photographed by fluorescence microscope.

### 4.6. Acridine Orange Staining

The zebrafish embryos were stained with 2 mg/L AO dye, and stained at room temperature for 20 min in the dark, and then washed twice with PBS solution. Zebrafish embryos were anesthetized with 0.05% Tricaine for 5 min. Apoptosis was observed in fluorescence microscopy and photographed.

### 4.7. Semi-Quantitative Polymerase Chain Reaction (PCR)

In this experiment, RNA was extracted by Trizol method. The purity and concentration of RNA samples were detected by gel electrophoresis and UV spectrophotometer and stored at −80 °C. PrimeScriptTM RT reagent Kit with gDNA Eraser was purchased from TaKaRa, Inc. The Premix rTag kit (TaKaRa) was used, using reverse-transcribed cDNA as the template, and the housekeeping gene used was ef1α. The primer sequences are shown in Table S1.

## Figures and Tables

**Table 1:** Statistics of Pb^2+^(0.4 mg/L) on spinal curvature of zebrafish in different periods.

Malformation	72 hpf	96 hpf	120 hpf
upward curvature	52.0 ± 1.5%	0%	0%
S-shaped curvature	0%	85.0 ± 2.5%	0%
downward curvature	0%	0%	100%
Total	52%	85%	100%

*n* = 50, repeat three times.

## Data Availability

Data are available on request due to restrictions, e.g., privacy or ethical. The data presented in this study are available on request from the corresponding author. The data are not publicly available as part of the data will be used for another unpublished article.

## Supplementary Materials

The following supporting information can be downloaded at: https://www.mdpi.com/article/10.3390/ijms23179571/s1.
