# Supplementary material for: Lead Exposure Causes Spinal Curvature during Embryonic Development in Zebrafish
Source: Int J Mol Sci. 2022 Aug 24;23(17):9571. doi: 10.3390/ijms23179571 (PMC9455242; doi:10.3390/ijms23179571)
Supplement: Supplementary file 1 [file ijms-23-09571-s001.zip › ijms-1876469-supplementary.pdf]

Supplementary Table

Table S1. Sequences of primers used in PCR.

Primers	Sequences
ef1a F	CCTGGGAGTGAAACAGCTGATC
ef1a R	CCGATCTTCTTGATGTATGCGCTG
bmp2b F	GGTGCCGTTGGACTCATT
bmp2b R	ACCACTGCCGATTTGCTT
runx2b F	CGGTGTCGGTGAAGATGA
runx2b R	AAAACGGAAGAGTAACCTCC
Shh F	AGACAAGAGCAAATACGGGACA
Shh R	AGCCGAACCTGGGAAACA
Ihh F	GCTCACGCCGAACTACAA
Ihh R	TGCCGTCTTCATCCCAAC
Bax F	GGAGGCGATACGGGCAGTG
Bax R	TTGCGAATCACCAATGCTGTG
Bcl2 F	TCGTTCAGACCCTCATTT
Bcl2 R	CATCCCAACCTCCATTTT
Perk F	GGACAACGTCGGAGGAGCCC
Perk R	GGCTAATGGGTGTCACCTTTCC
Chop F	GGATAGCTGATTGGTGCG
Chop R	CTCGTTTTGATCCGTCAGCTC
